# The Function and Roles of ADAMTS-7 in Inflammatory Diseases

**DOI:** 10.1155/2015/801546

**Published:** 2015-11-30

**Authors:** Yuying Zhang, Jiqiang Lin, Fanhua Wei

**Affiliations:** ^1^School of Biological Science and Technology, University of Jinan, Jinan 250022, China; ^2^Department of Pathology, New York University School of Medicine, New York, NY 10016, USA; ^3^College of Agriculture, Ningxia University, Yinchuan 750021, China

## Abstract

The ADAMTS proteinases are a group of multidomain and secreted metalloproteinases containing the thrombospondin motifs. ADAMTS-7 is a member of ADAMTS family and plays a crucial role in the pathogenesis of arthritis. Overexpression of ADAMTS-7 gene promotes the breakdown of cartilage oligomeric matrix protein (COMP) matrix and accelerates the progression of both surgically induced osteoarthritis and collagen-induced arthritis. Moreover, ADAMTS-7 and tumor necrosis factor-*α* (TNF-*α*) form a positive feedback loop in osteoarthritis. More significantly, granulin-epithelin precursor, a growth factor has important roles in bone development and bone-associated diseases, disturbs the interaction between ADAMTS-7 and COMP, and prevents COMP degradation. This review is based on our results and provides an overview of current knowledge of ADAMTS-7, including its structure, function, gene regulation, and inflammatory diseases involvement.

## 1. An Introduction to ADAMTS Family of Proteinases

The family of ADAMTS (a disintegrin and metalloproteinase with thrombospondin-like motifs) proteinases consists of 19 secreted, multidomain proteolytic enzymes and plays a crucial role in several pathophysiological processes including extracellular matrix (ECM) assembly and degradation, hemostasis, organogenesis, angiogenesis, genetic diseases, cancer, and arthritis [1]. The ADAMTS gene was first cloned as inflammation-associated gene in mice containing the TSP type I motif [2]. In general, the structure of ADAMTS proteins comprises a prodomain, a metalloproteinase domain, a disintegrin-like and spacer domain, and thrombospondin (TS) repeats [3]. The human ADAMTS proteins can be divided into four subgroups according to the sequence alignments and functional difference [4]. The first subgroup contains ADAMTS-1, -4, -5, -8, -9, -15, and -20 and degrades aggrecan. ADAMTS-2, -3, and -14 consist of the second subgroup and degrade peptides of procollagen [5–8]. ADAMTS-13 alone represents the third subgroup and is essential for von Willebrand factor cleavage (vWF) [9]. ADAMTS-7 and -12 that specifically associate with and degrade cartilage oligomeric matrix protein (COMP) belong to the fourth subgroup [10–13]. The detailed biological characteristics of ADAMTS proteins are summarized in Table 1.

## 2. ADAMTS-7

### 2.1. Structure

**Figure 1:**
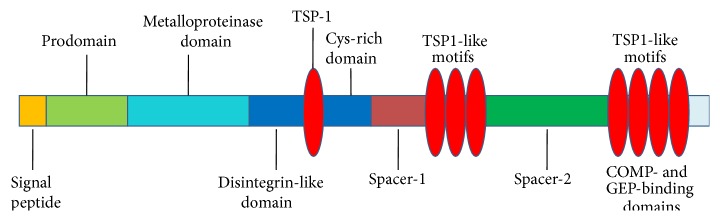
Domain structure and organization of ADAMTS-7.

As shown in Figure 1, ADAMTS-7 is a proteolytic member of the ADAMTS family comprising a signal peptide, a prodomain, a metalloproteinase domain, a disintegrin-like domain, and several thrombospondin type I repeats (TSP1), interspaced by spacer domains [14, 15]. The prodomain is generally considered to be essential for maintaining enzyme latency. Cleavage of the ADAMTS propeptide by convertases (furin or furin-like enzymes) is typically required for enzyme activity. For example, furin is the main convertase required for the maturation of ADAMTS-7 as compared with PACE4, PC6B, and PC7 convertases [13]. A metalloproteinase catalytic domain has a high degree sequence similarity of reprolysin-type zinc-binding motif, HEXXHXXG/N/SXXHD, and a methionine residue-containing Met-turn which plays an important role in the structure of the active site [16]. *α*2-Macroglobulin (*α*2M) associates with ADAMTS-7 and is a novel substrate of ADAMTS-7 [10], and the metalloproteinase domain of ADAMTS-7 alone is essential for cleavage of *α*2M [15]. The catalytic domain is also responsible for digestion of COMP at more than one site [14]. The disintegrin-like domain has a sequence similarity to the soluble snake venom disintegrins and may serve a function in regulating activity through providing an essential binding surface for substrates [17]. The C-terminal TS repeats between the disintegrin-like domain and cysteine-rich domain (CRD) of ADAMTS proteins are variable and interspaced by spacer domains; for instance, ADAMTS-4 lacks TS repeats [18], whereas ADAMTS-7 and ADAMTS-20 have four and fourteen TS repeats, respectively [14, 19]. The four C-terminal TS repeats of ADAMTS-7 are required and sufficient for interaction with EGF domains of COMP substrate and each granulin (GRN) unit of progranulin (PGRN) [10, 12, 20]. The spacer domain is the least homologous domain and in combination with a mucin domain between the third and fourth C-terminal TS repeats [13]. Unlike other ADAMTS proteins, the function of spacer domain appears not to be essential for interaction with ADAMTS-7 substrates, but for involvement in location of the enzyme [15].

### 2.2. Regulation of ADAMTS-7

ADAMTS-7 was expressed in bone, cartilage, synovium, tendon, and ligament, all of which contain COMP [12, 14]. ADAMTS-7 was also detectable in meniscus, skeletal muscle, and fat tissue [12, 14].* ADAMTS-7* mRNA transcripts of 8.0 kb and 4.5 kb were detected in skeletal muscle [15]. The identification of splice variants of ADAMTS-7 suggested that a potential posttranscriptional regulation might be a mechanism for gene regulation of ADAMTS-7. For example, miR-29a/b served a function in ADAMTS-7 mediated COMP degradation and subsequent vascular smooth muscle cells (VSMCs) calcification through directly targeting the 3′ untranslated region of ADAMTS-7 and markedly inhibited high-phosphate-induced ADAMTS-7 expression [21]. Furthermore, the ADAMTS-7 protein was also regulated at posttranslational levels since the recombinant protein from HEK293 stable lines showed a larger molecular weight [15]. Anyway, the physiological functions of ADAMTS-7 gene, which is expressed in many tissues at a basal level, need to be further elucidated.

TNF-*α* and interleukin- (IL-) 1*β* strongly upregulated the mRNA expression of ADAMTS-7 in human cartilage explants cultures [10]. Furthermore, the upregulation of ADAMTS-7 was also associated with the increased level of TNF-*α* in rheumatoid arthritis (RA) patients [12] and patients with femoral neck fracture (FNF) and osteonecrosis of femoral head (ONFH) at different stages [22]. Interestingly, TNF-*α* also induced the expression of ADAMTS-7, and the binding sites of inflammatory transcription factors NF-*κ*B and AP-1 were identified in the promoter of ADAMTS-7 gene by chromatin immunoprecipitation (ChIP) [23]. Our* in vivo* results also supported the relationship of ADAMTS-7 and TNF-*α*. Briefly, the surgically induced osteoarthritis (OA) model was established using* ADAMTS-7* transgenic mice and ADAMTS-7 small interfering (si)RNA knockdown mice; the results demonstrated that TNF-*α* activates the expression of ADAMTS-7 through NF-*κ*B mediator and ADAMTS-7 upregulates TNF-*α* and forms a positive loop between ADAMTS-7 and TNF-*α* in the pathogenesis of OA [24, 25].

### 2.3. The Role of ADAMTS-7 in Inflammatory Diseases

#### 2.3.1. Arthritis

Cells in all tissues are surrounded by extracellular matrix (ECM). ECM has an important role in providing structural support as a scaffold and regulating the cell activity and behavior, including cell shape, survival, differentiation, proliferation, and cell death [17]. The progression of arthritic diseases is characterized by the breakdown the ECM components and subsequent loss of articular cartilage and bone. COMP is a 524 kDa disulfide-bonded, multidomain glycoprotein composed of five 110 kDa subunits. COMP constitutes approximately 1% of the wet weight of the cartilage tissue and is a prominent noncollagenous component of cartilage ECM [26]. Mutations in the human COMP gene in a region that encoding the calmodulin-like repeat elements had been linked to the development of pseudoachondroplasia (PSACH) and multiple epiphyseal dysplasia (MED), which were dominantly inherited chondrodysplasias characterized by short stature and early-onset osteoarthrosis [27–30]. The pathophysiological function of COMP may be related to stabilizing the ECM of articular cartilage through interaction with matrix components such as collagen types II and IX, aggrecan, and fibronectin [31–34]. Degradative fragments of COMP had been observed in diseased cartilage, synovial fluid, and serum of patients with posttraumatic knee injuries, primary osteoarthritis (OA), and rheumatoid arthritis (RA) [35, 36]. Thus, the isolation of COMP-degradative enzymes is of great significance from both a pathophysiological mechanism and a therapeutic standpoint [14].

Several matrix metalloproteinases (MMP) can digest purified COMP* in vitro*, including MMP-1, MMP-3, MMP-9, MMP-13, MMP-19, and MMP-20 [37, 38]. In addition, ADAMTS-4 proteinase also can cleave COMP protein* in vitro* [39]. In these assays, the concentration of degradative enzymes and substrates is higher than physiological and pathological conditions. Furthermore, the exact role of ADAMTS proteinases in COMP degradation still needs to be further elucidated by* in vivo* animal studies. ADAMTS-7 and ADAMTS-12 were identified as the physiological enzymes responsible for COMP degradation by a functional genomic study [11, 12]. The interaction between ADAMTS-7 and COMP* in vitro* was verified using a glutathione S-transferase (GST) pulldown assay, and the specifically binding between ADAMTS-7 and COMP* in vivo* was further confirmed by coimmunoprecipitation assay. ADAMTS-7 colocalized with COMP both in the cytoplasm and on the surface of human chondrocytes and selectively interacted with the EGF repeat domain of COMP, whereas the four C-terminal TSP motifs of ADAMTS-7 were essential for association with COMP [12], supporting the notion that C-terminal domain of metalloproteinases are important for determining substrate specificity [40].

The recombinant enzyme of ADAMTS-7 purified from condition medium is able to digest COMP* in vitro*. The catalytic domain of ADAMTS-7 produced in transgenic bacteria as a GST fusion protein also can digest COMP in a time-dependent manner [12]. Intriguingly, the catalytic domain alone can degrade COMP and produce three fragments, suggesting that ADAMTS-7 might digest COMP at more than one site [12]. Furthermore, ADAMTS-7 was also involved in inflammatory cytokines TNF-*α*- and IL-1*β*-mediated digestion of COMP protein, whereas anti-ADAMTS-7 antibody efficiently blocked the production of 110 kDa COMP fragments [10]. These findings had been further verified using small interfering RNA silencing of ADAMTS-7 in human chondrocytes. Animal results from surgically induced OA and collagen-induced arthritis models using ADAMTS-7 transgenic mice also supported the digestion of COMP by ADAMTS-7* in vivo*. Overexpression of ADAMTS-7 in chondrocytes led to increasing COMP degradation in cartilage tissues using immunohistochemistry and significantly elevating serum levels of COMP proteolytic fragments by a novel sandwich enzyme linked immunosorbent assay (ELISA) [25] which is able to recognize epitopes of the COMP protein prone to degradation during the cartilage destruction [41]. The COMP fragments in joint sections and serum were significantly higher in collagen-induced arthritic ADAMTS-7 transgenic mice than that of the arthritic wild type controls [42].

No evident differences in ADAMTS-7 gene expression was observed between normal and OA patients' tissues [14]. However, ADAMTS-7 mRNA was found to be significantly increased in cartilage and synovium tissues from RA patients. The increasing COMP fragments were observed in cartilage, synovial fluid, and serum of OA and RA patients. And the COMP fragments degraded by recombinant ADAMTS-7 enzyme have a similar size to those seen in OA patients [10]. These findings suggested that the COMP degradation observed in OA and RA patients might associate with upregulation of ADAMTS-7.

Real-time PCR results of micromass cultures of a mouse embryonic mesenchymal stem cell line suggested that ADAMTS-7 was strongly induced during the terminal differentiation of chondrogenesis [43]. ADAMTS-7 was also highly expressed in both the early and later stages of cartilage development, as well as in chondrocytes throughout the mature growth plate [43]. These findings suggested that ADAMTS-7 may play a crucial role in chondrogenesis and may regulate various stages of cartilage development. Overexpression of ADAMTS-7 in murine mesenchymal stem cells resulted in efficient inhibition of chondrocyte differentiation, specifically during the stage of chondrocyte hypertrophy [43]. And the inhibitory effect of ADAMTS-7 on chondrocyte differentiation and endochondral bone growth was associated with inactivating granulin-epithelin precursor (GEP) and regulated by parathyroid hormone-related peptide (PTHrP) signaling [43]. Granulin epithelin precursor (GEP), also known as progranulin (PGRN), PC-cell-derived growth factor (PCDGF), proepithelin, and acrogranin, is a 593-amino-acid secreted growth factor [44, 45]. GEP contains seven-and-a-half repeats of a cysteine-rich motif in the order P-G-F-B-A-C-D-E [46]. GEP was highly expressed in chondrocytes of the musculoskeletal system [47] and played a key role in musculoskeletal development and diseases [48]. Recent reports suggested that GEP played a protective role in surgically induced OA [49, 50] and inflammatory arthritis [46]. Recombinant GEP decreased destruction of cartilage matrix and protected against OA progression in surgically induced OA models [49]. Moreover, PGRN was also involved in BMP-2 induction of osteoblastogenesis and ectopic bone formation [50]. PGRN-deficient mice were more susceptible to collagen-induced arthritis, and administration of PGRN reversed inflammatory arthritis through the inhibition of TNF-*α* signaling [46]. Data from yeast-2-hybrid and coimmunoprecipitation assays demonstrated that ADAMTS-7 binds to GEP [43]. GEP colocalized with ADAMTS-7 on the surface of chondrocytes and inhibited COMP degradation by ADAMTS-7 in a dose-dependent manner [20]. Intact GEP had anti-inflammatory effect through the inhibition of some of the actions of tumor necrosis factor, while the proteolytic peptides of GEP exerted proinflammatory effect through stimulating the production of proinflammatory cytokines such as interleukin-8 [51]. However, ADAMTS-7 also exerted its function as a GEP convertase and was involved in the proteolytic processing of GEP with the production of small fragments [43]. Overall, ADAMTS-7 metalloproteinases, COMP matrix protein, GEP growth factor, and TNF-*α* inflammatory cytokine all act in concert to form a key interaction and interplay networks in the pathogenesis of arthritis.

**Figure 2:**
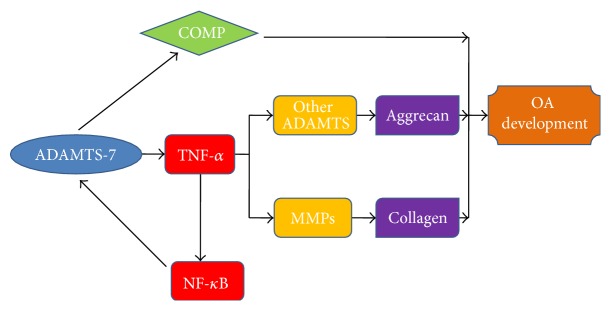
A proposed model for the potential role and mechanism of ADAMTS-7 in the regulation of OA development (edited according to [25]).

**Figure 3:**
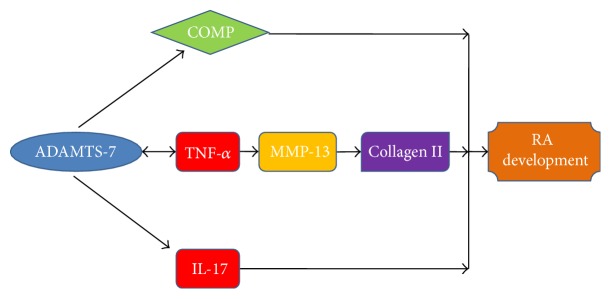
A proposed model for the potential role and regulation of ADAMTS-7 in the pathogenesis of inflammatory arthritis (edited according to [42]).

In order to further elucidate the role of ADAMTS-7 in cartilage development and endochondral bone growth* in vivo*, the transgenic mice were generated through targeting overexpression of ADAMTS-7 in chondrocytes regulated by Col II promoter, and knockdown mice were generated using Cre/loxp system [25]. Targeted overexpression of ADAMTS-7 in chondrocytes resulted in chondrodysplasia characterized by short-limbed dwarfism and a delay in endochondral ossification in “young mice” and a spontaneous OA-like phenotype in “aged” mice [25]. In surgically induced OA model, evident cartilage loss was found in transgenic mice at 4 weeks after surgery, whereas moderate cartilage loss was observed in wild type mice at 8 weeks after surgery. However, no evident cartilage loss occurred in ADAMTS-7 small interfering (si)RNA knockdown mice even at 12 weeks after surgery [25]. Anyway, these findings suggested that overexpression of ADAMTS-7 exaggerated destruction of cartilage and accelerated development of OA, while knockdown of ADAMTS-7 attenuated breakdown of cartilage matrix and protects against OA progression. The potential mechanism of ADAMTS-7 in the regulation of OA progression is summarized in Figure 2. In collagen-induced arthritis (CIA) mode, ADAMTS-7 transgenic mice were more susceptible to induction of CIA, and arthritic transgenic mice displayed significantly higher clinical and histological arthritis scores as compared with wild type mice [42]. The role of ADAMTS-7 in the pathogenesis of collagen-induced inflammatory arthritis was also summarized in Figure 3. Thus, ADAMTS-7 expression was elevated during disease progression in surgically induced OA and collagen-induced arthritis model, and the increasing ADAMTS-7 upregulated the level of inflammatory cytokines including TNF-*α* [24, 25]. The elevated expression of ADAMTS-7 led to accelerated degradation of COMP. In addition, the upregulation of inflammatory cytokine TNF-*α* induced the expression of MMP and other ADAMTS members. Eventually, these factors resulted in accelerated progression of arthritis [24, 25]. Collectively, the role of ADAMTS-7 in the pathogenesis of arthritis is associated with degradation of COMP and upregulation of inflammatory cytokines and other metalloproteinases.

In addition, ADAMTS-12 also played a critical role in the pathogenesis of arthritis since ADAMTS-7 and ADAMTS-12 share the common substrate (COMP) [11]. The expression of ADAMTS-12 was significantly increased in the cartilage and synovium of OA or RA patients [52, 53]. ADAMTS-12 expression is required for normal cartilage development and its dysregulation results in defects in the musculoskeletal system including brachydactyly type E (BDE) [54]. The potential role of ADAMTS-7 in OA is related to association and degradation of COMP matrix [14, 54]. ADAMTS-12 as an inflammatory protein and also played a role in RA [55]. The genotyping results of three single nucleotide polymorphisms (SNPs) of ADAMTS-12 in 303 RA patients and 495 control subjects suggested that the genotype frequency of rs10461703 was associated with the RA development [55]. Overall, ADAMTS-12 has an essential role in the progression of arthritis and may serve as a therapeutic target for arthritis treatments. And results from ADAMTS-12 mice are helpful for investigating its exact role in arthritic conditions.

#### 2.3.2. Atherosclerosis

Atherosclerosis is a progressive inflammatory disease triggered by damage to the vascular endothelium by many risk factors such as genetic predisposition, hypertension, and type 2 diabetes mellitus [56]. The inflammatory process ultimately leads to the development of complex plaques composed of cholesterol, lipids, inflammatory cells, and debris resulting from cell apoptosis [56, 57]. ADAMTS proteinases and their ability to interaction with ECM have been implicated in the pathogenesis of vascular disease processes including atherosclerosis. These disease processes characterize by media-to-intima migration of vascular smooth muscle cells (VSMCs), resulting in thickening of the intimal layer of vessel [58–60]. The matrix metalloproteinase-mediated degradation and remodeling of ECM plays an essential role in these disease processes and form a barrier to VSMC migration [61]. In atherosclerosis progression, macrophages and monocytes secrete the ADAMTS proteinases to influence the stability of the complex plaque [62]. Several ADAMTS members were highly expressed in human carotid lesions and advanced coronary atherosclerotic plaques, including ADAMTS-1, -4, -5, and -8 [63]. In the mouse carotid artery flow cessation model, ADAMTS-1 transgenic/apoE-deficient mice show a significant increase in intimal hyperplasia as compared with apoE-deficient mice [64]. These findings suggested that the potential role of ADAMTS proteinases in atherosclerosis might associate with accelerated degradation of ECM of vessel.

Results from genome-wide association studies (GWAS) demonstrated that ADAMTS-7 was tightly associated with the development of coronary atherosclerosis in existing coronary atherosclerosis [65–68]. A common SNP near ADAMTS-7 was a common genetic risk factor for coronary atherosclerosis, with a 19% increased risk for carriers [69]. The casual link between ADAMTS-7 and atherosclerosis progression has yet to be established. Neointima formation is considered as a response to vessel injury. The ADAMTS-7 protein was expressed preferentially in neointima of the carotid artery wall in response to balloon injury and colocalized with VSMCs in the newly formed neointima [70–72]. The augmented expression of ADAMTS-7 increased the proliferation and migration of VSMCs, while suppression of ADAMTS-7 level using small interfering RNA (siRNA) had the opposite effect in the rat model. The notion was supported by the results from knockout mice mode which demonstrate that ADAMTS-7 deficiency led to reduce neointima formation following carotid artery injury induced by ligation [73]. These findings suggested that ADAMTS-7 had a critical role in intimal hyperplasia after vascular injury.

COMP, a component of vascular ECM which has been observed in atherosclerotic lesions, is thought to be involved in migration of VSMCs [74]. Overexpression of COMP markedly inhibited VSMC dedifferentiation and the expression of phenotype-dependent markers [23], while knockdown of ADAMTS-7 evidently attenuated COMP degradation and retarded VSMCs calcification [21], suggesting that the ADAMTS-7-mediated migration of VSMCs might associate with degradation of COMP matrix. However, ADAMTS-7 also can bind directly to thrombospondin-1 (TSP-1) and be involved in endothelium repair through COMP-independent pathways since COMP deficiency did not affect reendothelialization in injured arteries [75]. These findings suggested that ADAMTS-7 is a potential therapeutic target for atherosclerosis and vascular disorders [23, 66, 75, 76]. In conclusion, ADAMTS-7 is involved in the pathogenesis of vascular disorders through degradation of COMP matrix and TSP-1, accelerated migration and proliferation of VSMCs, and regulation of inflammatory cytokines.

#### 2.3.3. Other Pathological Conditions

ADAMTS-7 as a connective tissue growth factor (CTGF) binding and processing protein and has been reported to be an important regulator in oval cell (OC) activation and biliary fibrosis, and its deficiency decreased CTGF turnover ability and enhanced hepatic progenitor/oval cell (HPC/OC) activation and biliary fibrosis during 3,5-diethoxycarbonyl-1,4-dihydrocollidine- (DDC-) induced liver injury [77]. ADAMTS-7 expression had been found in urine from patients with prostate, bladder, and breast cancer, suggesting a diagnostic and prognostic role of ADAMTS-7 in the detection and therapeutic value in tumor growth, invasion, and metastasis [78]. In addition, ADAMTS-7 was also found to be involved in host-pathogen interaction [79, 80]. ADAMTS-7 played a critical role in influenza virus replication and was involved in host cell pathways such as NF-*κ*B activation, and its gene expression resulted in reduced influenza virus replication through inhibition of miR-106B [79]. ADAMTS-7 had been addressed to be involved into* V. splendidus* challenged sea cucumber and had significantly global proteome changes in expression at all examined time points using isobaric tags for relative and absolute quantification (iTRAQ) as compared with control group [80].

## 3. Conclusion

ADAMTS-7 is a member of the ADAMTS family, which are a group of secreted enzymes containing 19 members. The ADAMTS proteinase members play a key role in a variety of pathophysiological processes including development, human genetic diseases, and chronic inflammatory conditions. In the present review, we focused on the role of ADAMTS-7 in the progression of inflammatory diseases including arthritis and atherosclerosis. Overexpression of ADAMTS-7 accelerated the degradation of COMP and the onset and progression of arthritis through formation of a positive feedback loop with TNF-*α*. ADAMTS-7 has potential to serve as a therapeutic drug target in arthritis conditions. To do so, the precise understanding of the exact role played by ADAMTS-7 and its binding partners in inflammatory diseases appears to be of particular importance.

## Figures and Tables

**Table 1:** Biological characteristics of ADAMTS family members.

Gene	Proteolytic activity	Expression in human tissues	Substrates	Role	References
*ADAMTS-1*	+	Liver, endotheliocyte, skeletal muscle, and ovary	Aggrecan, versican	Cancer, atherosclerosis, fibrosis, antiangiogenesis, ovarian function, and stress	[81–89]

*ADAMTS-2*	+	Connective tissue, placenta	Procollagen	Ehlers-Danlos syndrome, mesothelioma, and placenta development	[90–93]

*ADAMTS-3*	+	Skin, lung, and brain	Procollagen	Dermatosparaxis, osteoarthritis, and lymphangiogenesis	[7, 94, 95]

*ADAMTS-4*	+	Heart, lung, skeletal muscle, liver, and kidney	Aggrecan, COMP, and brevican	Glioma, atherosclerosis, arthritis, and tendinopathy	[63, 96–99]

*ADAMTS-5*	+	Macrophage, bladder, oesophagus, and heart	Aggrecan, brevican	Arthritis, cancer	[96, 100, 101]

*ADAMTS-6*				Tissue repair	[102]

*ADAMTS-7*	+	Heart, liver, kidney, and skeletal muscle	COMP, *α*2M	Arthritis, atherosclerosis, and kidney damage	[14, 42, 70, 103]

*ADAMTS-8*	+	Heart, lung, and kidney	Aggrecan	Cancer, atherosclerosis, arthritis, and antiangiogenesis	[63, 104–106]

*ADAMTS-9*	+	Heart, lung, and skeletal muscle	Aggrecan, versican	Cancer, atherosclerosis, arthritis, and tissue syndactyly	[107–109]

*ADAMTS-10*		Lens, cartilage, and skin		Weill-Marchesani syndrome	[110]

*ADAMTS-12*	+	Chondrocyte, lung, kidney, and liver	COMP, *α*2M, and aggrecan	Arthritis, cancer, and normal inflammatory response	[14, 54, 111–113]

*ADAMTS-13*	+	Liver, placenta, heart, and skeletal muscle	von Willebrand factor (vWf)	Thrombotic thrombocytopenic purpura	[9]

*ADAMTS-14*	+	Collagen-rich tissue, lung, and kidney	Procollagen	Fibrosis, osteoarthritis, tendon disorders, and sclerosis	[114–118]

*ADAMTS-15*	+	Kidney, lung, heart, ovary, and stem cells	Aggrecan	Cancer, follicle rupture, myogenesis, and spinal injury	[1, 119–122]

*ADAMTS-16*	+	Lung, kidney, ovary, cartilage, and brain	*α*2M	Cancer, cryptorchidism, and premature ovarian failure	[22, 123–125]

*ADAMTS-17*		Epidermis, brain, heart, liver, lung, and prostate		Weill-Marchesani syndrome, short stature, and pediatric stroke	[126–128]

*ADAMTS-18*		Lung, kidney, liver, brain, and prostate chondrocyte		Ocular disease, cancer, stroke, and bone disorders	[129–133]

*ADAMTS-19*		Lung, endothelium, ovary	Aggrecan	Premature ovarian failure, osteosarcomas	[134–137]

*ADAMTS-20*		Ovary, heart, lung, placenta, and testis	Aggrecan	Melanocyte differentiation, pigmentation, and apoptosis	[19, 138]

## Abbreviations

ADAMTS: A disintegrin and metalloproteinase with thrombospondin motifs
TNF-*α*: Tumor necrosis factor-*α*
COMP: Cartilage oligomeric matrix protein
ECM: Extracellular matrix
TSP: Thrombospondin
vWF: von Willebrand factor
*α*2-M: *α*2-Macroglobulin
CRD: Cysteine-rich domain
VSMCs: Vascular smooth muscle cells
RA: Rheumatoid arthritis
FNF: Femoral neck fracture
ONFH: Osteonecrosis of femoral head
GEP: Granulin-epithelin precursor
MED: Multiple epiphyseal dysplasia
ELISA: Enzyme linked immunosorbent assay
MMP: Matrix metalloproteinases
PCDGF: PC-cell-derived growth factor
PTHrP: Parathyroid hormone-related peptide
GWAS: Genome-wide association studies.

## References

[1] Wagstaff L., Kelwick R., Decock J., Edwards D. R. (2011). The roles of ADAMTS metalloproteinases in tumorigenesis and metastasis. *Frontiers in Bioscience*.

[2] Kuno K., Kanada N., Nakashirma E., Fujiki F., Ichimura F., Matsushima K. (1997). Molecular cloning of a gene encoding a new type of metalloproteinase-disintegrin family protein with thrombospondin motifs as an inflammation associated gene. *Journal of Biological Chemistry*.

[3] Nicholson A. C., Malik S.-B., Logsdon J. M., Van Meir E. G. (2005). Functional evolution of ADAMTS genes: evidence from analyses of phylogeny and gene organization. *BMC Evolutionary Biology*.

[4] Jones G. C., Riley G. P. (2005). ADAMTS proteinases: a multi-domain, multi-functional family with roles in extracellular matrix turnover and arthritis. *Arthritis Research and Therapy*.

[5] Colige A., Li S.-W., Sieron A. L., Nusgens B. V., Prockop D. J., Lapière C. M. (1997). cDNA cloning and expression of bovine procollagen I N-proteinase: a new member of the superfamily of zinc-metalloproteinases with binding sites for cells and other matrix components. *Proceedings of the National Academy of Sciences of the United States of America*.

[6] Wang W.-M., Lee S., Steiglitz B. M. (2003). Transforming growth factor-*β* induces secretion of activated ADAMTS-2. A procollagen III N-proteinase. *Journal of Biological Chemistry*.

[7] Fernandes R. J., Hirohata S., Engle J. M. (2001). Procollagen II amino propeptide processing by ADAMTS-3. Insights on dermatosparaxis. *Journal of Biological Chemistry*.

[8] Colige A., Vandenberghe I., Thiry M. (2002). Cloning and characterization of ADAMTS-14, a novel ADAMTS displaying high homology with ADAMTS-2 and ADAMTS-3. *Journal of Biological Chemistry*.

[9] Soejima K., Matsumoto M., Kokame K. (2003). ADAMTS-13 cysteine-rich/spacer domains are functionally essential for von Willebrand factor cleavage. *Blood*.

[10] Luan Y., Kong L., Howell D. R. (2008). Inhibition of ADAMTS-7 and ADAMTS-12 degradation of cartilage oligomeric matrix protein by *α*-2-macroglobulin. *Osteoarthritis and Cartilage*.

[11] Liu C.-J., Kong W., Xu K. (2006). ADAMTS-12 associates with and degrades cartilage oligomeric matrix protein. *The Journal of Biological Chemistry*.

[12] Liu C.-J., Kong W., Ilalov K. (2006). ADAMTS-7: a metalloproteinase that directly binds to and degrades cartilage oligomeric matrix protein.. *The FASEB Journal*.

[13] Somerville R. P. T., Longpré J.-M., Apel E. D. (2004). ADAMTS7B, the full-length product of the ADAMTS7 gene, is a chondroitin sulfate proteoglycan containing a mucin domain. *The Journal of Biological Chemistry*.

[14] Liu C.-J. (2009). The role of ADAMTS-7 and ADAMTS-12 in the pathogenesis of arthritis. *Nature Clinical Practice Rheumatology*.

[15] Hanby H. A., Zheng X. L. (2013). Biochemistry and physiological functions of ADAMTS7 metalloprotease. *Advances in Biochemistry*.

[16] Porter S., Clark I. M., Kevorkian L., Edwards D. R. (2005). The ADMTS metalloproteinases. *Biochemical Journal*.

[17] Stanton H., Melrose J., Little C. B., Fosang A. J. (2011). Proteoglycan degradation by the ADAMTS family of proteinases. *Biochimica et Biophysica Acta*.

[18] Mosyak L., Georgiadis K., Shane T. (2008). Crystal structures of the two major aggrecan degrading enzymes, ADAMTS4 and ADAMTS5. *Protein Science*.

[19] Llamazares M., Cal S., Quesada V., López-Otín C. (2003). Identification and characterization of ADAMTS-20 defines a novel subfamily of metalloproteinases-disintegrins with multiple thrombospondin-1 repeats and a unique GON domain. *The Journal of Biological Chemistry*.

[20] Guo F., Lai Y., Tian Q., Lin E. A., Kong L., Liu C. (2010). Granulin-epithelin precursor binds directly to ADAMTS-7 and ADAMTS-12 and inhibits their degradation of cartilage oligomeric matrix protein. *Arthritis & Rheumatism*.

[21] Du Y., Gao C., Liu Z. (2012). Upregulation of a disintegrin and metalloproteinase with thrombospondin motifs-7 by miR-29 repression mediates vascular smooth muscle calcification. *Arteriosclerosis, Thrombosis, and Vascular Biology*.

[22] Abdul-Majeed S., Mell B., Nauli S. M., Joe B. (2014). Cryptorchidism and infertility in rats with targeted disruption of the Adamts16 locus. *PLoS ONE*.

[23] Wang L., Zheng J., Du Y. (2010). Cartilage oligomeric matrix protein maintains the contractile phenotype of vascular smooth muscle cells by interacting with *α*7*β*1 integrin. *Circulation Research*.

[24] Buckland J. (2013). Osteoarthritis: positive feedback between ADAMTS-7 and TNF in OA. *Nature Reviews Rheumatology*.

[25] Lai Y., Bai X., Zhao Y. (2014). ADAMTS-7 forms a positive feedback loop with TNF-alpha in the pathogenesis of osteoarthritis. *Annals of the Rheumatic Diseases*.

[26] Hedbom E., Antonsson P., Hjerpe A. (1992). Cartilage matrix proteins. An acidic oligomeric protein (COMP) detected only in cartilage. *Journal of Biological Chemistry*.

[27] Briggs M. D., Huffman S. M. G., King L. M. (1995). Pseudoachondroplasia and multiple epiphyseal dysplasia due to mutations in the cartilage oligomeric matrix protein gene. *Nature Genetics*.

[28] Briggs M. D., Mortier G. R., Cole W. G. (1998). Diverse mutations in the gene for cartilage oligomeric matrix protein in the pseudoachondroplasia-multiple epiphyseal dysplasia disease spectrum. *The American Journal of Human Genetics*.

[29] Song H.-R., Lee K.-S., Li Q.-W., Koo S. K., Jung S.-C. (2003). Identification of cartilage oligomeric matrix protein (COMP) gene mutations in patients with pseudoachondroplasia and multiple epiphyseal dysplasia. *Journal of Human Genetics*.

[30] Cohn D. H., Briggs M. D., King L. M. (1996). Mutations in the cartilage oligomeric matrix protein (COMP) gene in pseudoachondroplasia and multiple epiphyseal dysplasia. *Annals of the New York Academy of Sciences*.

[31] Chan I., Liu L., Hamada T., Sethuraman G., Mcgrath J. A. (2007). The molecular basis of lipoid proteinosis: mutations in extracellular matrix protein 1. *Experimental Dermatology*.

[32] Di Cesare P. E., Chen F. S., Moergelin M. (2002). Matrix-matrix interaction of cartilage oligomeric matrix protein and fibronectin. *Matrix Biology*.

[33] Månsson B., Carey D., Alini M. (1995). Cartilage and bone metabolism in rheumatoid arthritis. Differences between rapid and slow progression of disease identified by serum markers of cartilage metabolism. *Journal of Clinical Investigation*.

[34] Rosenberg K., Olsson H., Mörgelin M., Heinegård D. (1998). Cartilage oligomeric matrix protein shows high affinity zinc-dependent interaction with triple helical collagen. *The Journal of Biological Chemistry*.

[35] Neidhart M., Hauser N., Paulsson M., Dicesare P. E., Michel B. A., Häuselmann H. J. (1997). Small fragments of cartilage oligomeric matrix protein in synovial fluid and serum as markers for cartilage degradation. *British Journal of Rheumatology*.

[36] Saxne T., Heinegard D. (1992). Cartilage oligomeric matrix protein: a novel marker of cartilage turnover detectable in synovial fluid and blood. *British Journal of Rheumatology*.

[37] Ganu V., Goldberg R., Peppard J. (1998). Inhibition of interleukin-1*α*-induced cartilage oligomeric matrix protein degradation in bovine articular cartilage by matrix metalloproteinase inhibitors: potential role for matrix metalloproteinases in the generation of cartilage oligomeric matrix protein fragments in arthritic synovial fluid. *Arthritis and Rheumatism*.

[38] Stracke J. O., Fosang A. J., Last K. (2000). Matrix metalloproteinases 19 and 20 cleave aggrecan and cartilage oligomeric matrix protein (COMP). *FEBS Letters*.

[39] Dickinson S. C., Vankemmelbeke M. N., Buttle D. J., Rosenberg K., Heinegård D., Hollander A. P. (2003). Cleavage of cartilage oligomeric matrix protein (thrombospondin-5) by matrix metalloproteinases and a disintegrin and metalloproteinase with thrombospondin motifs. *Matrix Biology*.

[40] Martel-Pelletier J., Welsch D. J., Pelletier J.-P. (2001). Metalloproteases and inhibitors in arthritic diseases. *Best Practice and Research: Clinical Rheumatology*.

[41] Lai Y., Yu X.-P., Zhang Y. (2012). Enhanced COMP catabolism detected in serum of patients with arthritis and animal disease models through a novel capture ELISA. *Osteoarthritis and Cartilage*.

[42] Zhang Y., Wei F., Liu C. J. (2015). Overexpression of ADAMTS-7 leads to accelerated initiation and progression of collagen-induced arthritis in mice. *Molecular and Cellular Biochemistry*.

[43] Bai X.-H., Wang D.-W., Kong L. (2009). ADAMTS-7, a direct target of PTHrP, adversely regulates endochondral bone growth by associating with and inactivating GEP growth factor. *Molecular and Cellular Biology*.

[44] Anakwe O. O., Gerton G. L. (1990). Acrosome biogenesis begins during meiosis: evidence from the synthesis and distribution of an acrosomal glycoprotein, acrogranin, during guinea pig spermatogenesis. *Biology of Reproduction*.

[45] Ong C. H. P., Bateman A. (2003). Progranulin (granulin-epithelin precursor, PC-cell derived growth factor, acrogranin) in proliferation and tumorigenesis. *Histology and Histopathology*.

[46] Tang W., Lu Y., Tian Q.-Y. (2011). The growth factor progranulin binds to TNF receptors and is therapeutic against inflammatory arthritis in mice. *Science*.

[47] Xu K., Zhang Y., Ilalov K. (2007). Cartilage oligomeric matrix protein associates with Granulin-Epithelin Precursor (GEP) and potentiates GEP-stimulated chondrocyte proliferation. *The Journal of Biological Chemistry*.

[48] Konopka J., Richbourgh B., Liu C. (2014). The role of PGRN in musculoskeletal development and disease. *Frontiers in Bioscience*.

[49] Zhao Y.-P., Liu B., Tian Q.-Y., Wei J.-L., Richbourgh B., Liu C.-J. (2014). Progranulin protects against osteoarthritis through interacting with TNF-*α* and *β*-catenin signalling. *Annals of the Rheumatic Diseases*.

[50] Zhao Y.-P., Tian Q.-Y., Frenkel S., Liu C.-J. (2013). The promotion of bone healing by progranulin, a downstream molecule of BMP-2, through interacting with TNF/TNFR signaling. *Biomaterials*.

[51] He Z., Bateman A. (2003). Progranulin (granulin-epithelin precursor, PC-cell-derived growth factor, acrogranin) mediates tissue repair and tumorigenesis. *Journal of Molecular Medicine*.

[52] Davidson R. K., Waters J. G., Kevorkian L. (2006). Expression profiling of metalloproteinases and their inhibitors in synovium and cartilage. *Arthritis Research and Therapy*.

[53] Kevorkian L., Young D. A., Darrah C. (2004). Expression profiling of metalloproteinases and their inhibitors in cartilage. *Arthritis & Rheumatism*.

[54] Wei J., Richbourgh B., Jia T., Liu C. (2014). ADAMTS-12: a multifaced metalloproteinase in arthritis and inflammation. *Mediators of Inflammation*.

[55] Nah S.-S., Lee S., Joo J. (2012). Association of ADAMTS12 polymorphisms with rheumatoid arthritis. *Molecular Medicine Reports*.

[56] Salter R. C., Ashlin T. G., Kwan A. P. L., Ramji D. P. (2010). ADAMTS proteases: key roles in atherosclerosis?. *Journal of Molecular Medicine*.

[57] Glass C. K., Witztum J. L. (2001). Atherosclerosis: the road ahead. *Cell*.

[58] Newby A. C., Zaltsman A. B. (2000). Molecular mechanisms in intimal hyperplasia. *The Journal of Pathology*.

[59] Davies M. G., Hagen P.-O. (1994). Pathobiology of intimal hyperplasia. *British Journal of Surgery*.

[60] Rudijanto A. (2007). The role of vascular smooth muscle cells on the pathogenesis of atherosclerosis. *Acta Medica Indonesiana*.

[61] Hu J., Van den Steen P. E., Sang Q.-X. A., Opdenakker G. (2007). Matrix metalloproteinase inhibitors as therapy for inflammatory and vascular diseases. *Nature Reviews Drug Discovery*.

[62] Worley J. R., Baugh M. D., Hughes D. A. (2003). Metalloproteinase expression in PMA-stimulated THP-1 cells: effects of peroxisome proliferator-activated receptor-*γ* (PPAR*γ*) agonists and 9-cis-retinoic acid. *The Journal of Biological Chemistry*.

[63] Wågsäter D., Björk H., Zhu C. (2008). ADAMTS-4 and -8 are inflammatory regulated enzymes expressed in macrophage-rich areas of human atherosclerotic plaques. *Atherosclerosis*.

[64] Jönsson-Rylander A.-C., Nilsson T., Fritsche-Danielson R. (2005). Role of ADAMTS-1 in atherosclerosis: remodeling of carotid artery, immunohistochemistry, and proteolysis of versican. *Arteriosclerosis, Thrombosis, and Vascular Biology*.

[65] van Setten J., Isgum I., Smolonska J. (2013). Genome-wide association study of coronary and aortic calcification implicates risk loci for coronary artery disease and myocardial infarction. *Atherosclerosis*.

[66] Reilly M. P., Li M., He J. (2011). Identification of ADAMTS7 as a novel locus for coronary atherosclerosis and association of ABO with myocardial infarction in the presence of coronary atherosclerosis: two genome-wide association studies. *The Lancet*.

[67] Newby A. C. (2014). Proteinases and plaque rupture: unblocking the road to translation. *Current Opinion in Lipidology*.

[68] You L., Tan L., Liu L. (2015). ADAMTS7 locus confers high cross-race risk for development of coronary atheromatous plaque. *Molecular Genetics and Genomics*.

[69] Lotta L. A., Peyvandi F. (2011). Addressing the complexity of cardiovascular disease by design. *The Lancet*.

[70] Wang L., Wang X., Kong W. (2010). ADAMTS-7, a novel proteolytic culprit in vascular remodeling. *Sheng Li Xue Bao*.

[71] Bauer R. C., Tohyama J., Cui J. (2015). Knockout of *Adamts7*, a novel coronary artery disease locus in humans, reduces atherosclerosis in mice. *Circulation*.

[72] Zhang L., Yu F., Wang L. (2015). ADAMTS-7 promotes vascular smooth muscle cells proliferation in vitro and in vivo. *Science China Life Sciences*.

[73] Patel R. S., Ye S. (2013). ADAMTS7: a promising new therapeutic target in coronary heart disease. *Expert Opinion on Therapeutic Targets*.

[74] Riessen R., Fenchel M., Chen H., Axel D. I., Karsch K. R., Lawler J. (2001). Cartilage oligomeric matrix protein (thrombospondin-5) is expressed by human vascular smooth muscle cells. *Arteriosclerosis, Thrombosis, and Vascular Biology*.

[75] Kessler T., Zhang L., Liu Z. (2015). ADAMTS-7 inhibits re-endothelialization of injured arteries and promotes vascular remodeling through cleavage of thrombospondin-1. *Circulation*.

[76] Arroyo A. G., Andrés V. (2015). ADAMTS7 in cardiovascular disease: from bedside to bench and back again?. *Circulation*.

[77] Pi L., Jorgensen M., Oh S. H. (2015). A disintegrin and metalloprotease with thrombospondin type I motif 7: a new protease for connective tissue growth factor in hepatic progenitor/oval cell niche. *The American Journal of Pathology*.

[78] Roy R., Louis G., Loughlin K. R. (2008). Tumor-specific urinary matrix metalloproteinase fingerprinting: identification of high molecular weight urinary matrix metalloproteinase species. *Clinical Cancer Research*.

[79] Meliopoulos V. A., Andersen L. E., Brooks P. (2012). MicroRNA regulation of human protease genes essential for influenza virus replication. *PLoS ONE*.

[80] Zhang P., Li C., Zhang P., Jin C., Pan D., Bao Y. (2014). iTRAQ-based proteomics reveals novel members involved in pathogen challenge in sea cucumber *Apostichopus japonicus*. *PLoS ONE*.

[81] Shindo T., Kurihara H., Kuno K. (2000). ADAMTS-1: a metalloproteinase-disintegrin essential for normal growth, fertility, and organ morphology and function. *Journal of Clinical Investigation*.

[82] Freitas V. M., do Amaral J. B., Silva T. A. (2013). Decreased expression of ADAMTS-1 in human breast tumors stimulates migration and invasion. *Molecular Cancer*.

[83] Nakamura A., Sakai Y., Ohata C., Komurasaki T. (2007). Expression and significance of a disintegrin and metalloproteinase with thrombospondin motifs (ADAMTS)-1 in an animal model of renal interstitial fibrosis induced by unilateral ureteral obstruction. *Experimental and Toxicologic Pathology*.

[84] Mittaz L., Ricardo S., Martinez G. (2005). Neonatal calyceal dilation and renal fibrosis resulting from loss of Adamts-1 in mouse kidney is due to a developmental dysgenesis. *Nephrology Dialysis Transplantation*.

[85] Vázquez F., Hastings G., Ortega M.-A. (1999). METH-1, a human ortholog of ADAMTS-1, and METH-2 are members of a new family of proteins with angio-inhibitory activity. *The Journal of Biological Chemistry*.

[86] Toms D., Xu S., Pan B., Wu D., Li J. (2015). Progesterone receptor expression in granulosa cells is suppressed by microRNA-378-3p. *Molecular and Cellular Endocrinology*.

[87] Hong-Brown L. Q., Brown C. R., Navaratnarajah M., Lang C. H. (2015). Adamts1 mediates ethanol-induced alterations in collagen and elastin via a FoxO1-sestrin3-ampk signaling cascade in myocytes. *Journal of Cellular Biochemistry*.

[88] Toms D., Xu S., Pan B., Wu D., Li J. (2015). Progesterone receptor expression in granulosa cells is suppressed by microRNA-378-3p. *Molecular and Cellular Endocrinology*.

[89] Hong-Brown L. Q., Brown C. R., Navaratnarajah M., Lang C. H. (2015). Adamts1 mediates ethanol-induced alterations in collagen and elastin via a FoxO1-sestrin3-ampk signaling cascade in myocytes. *Journal of Cellular Biochemistry*.

[90] Colige A., Sieron A. L., Li S.-W. (1999). Human ehlers-danlos syndrome type VII C and bovine dermatosparaxis are caused by mutations in the procollagen I N-proteinase gene. *American Journal of Human Genetics*.

[91] Dubail J., Kesteloot F., Deroanne C. (2010). ADAMTS-2 functions as anti-angiogenic and anti-tumoral molecule independently of its catalytic activity. *Cellular and Molecular Life Sciences*.

[92] Matullo G., Guarrera S., Betti M. (2013). Genetic variants associated with increased risk of malignant pleural mesothelioma: a genome-wide association study. *PLoS ONE*.

[93] Takahashi H., Yuge K., Matsubara S. (2014). Differential expression of ADAM (a disintegrin and metalloproteinase) genes between human first trimester villous and extravillous trophoblast cells. *Journal of Nippon Medical School*.

[94] Kawahara C., Forster T., Chapman K., Carr A., Loughlin J. (2005). Genetic association analysis of the IGFBP7, ADAMTS3, and IL8 genes as the potential osteoarthritis susceptibility that maps to chromosome 4q. *Annals of the Rheumatic Diseases*.

[95] Jeltsch M., Jha S. K., Tvorogov D. (2014). CCBE1 enhances lymphangiogenesis via A disintegrin and metalloprotease with thrombospondin motifs-3-mediated vascular endothelial growth factor-C activation. *Circulation*.

[96] Verma P., Dalal K. (2011). ADAMTS-4 and ADAMTS-5: key enzymes in osteoarthritis. *Journal of Cellular Biochemistry*.

[97] Majumdar M. K., Askew R., Schelling S. (2007). Double-knockout of ADAMTS-4 and ADAMTS-5 in mice results in physiologically normal animals and prevents the progression of osteoarthritis. *Arthritis and Rheumatism*.

[98] Glasson S. S., Askew R., Sheppard B. (2004). Characterization of and osteoarthritis susceptibility in ADAMTS-4-knockout mice. *Arthritis and Rheumatism*.

[99] Corps A. N., Jones G. C., Harrall R. L., Curry V. A., Hazleman B. L., Riley G. P. (2008). The regulation of aggrecanase ADAMTS-4 expression in human Achilles tendon and tendon-derived cells. *Matrix Biology*.

[100] Nakada M., Miyamori H., Kita D. (2005). Human glioblastomas overexpress ADAMTS-5 that degrades brevican. *Acta Neuropathologica*.

[101] Didangelos A., Mayr U., Monaco C., Mayr M. (2012). Novel role of ADAMTS-5 protein in proteoglycan turnover and lipoprotein retention in atherosclerosis. *The Journal of Biological Chemistry*.

[102] Tababat-Khani P., Berglund L. M., Agardh C.-D., Gomez M. F., Agardh E. (2013). Photocoagulation of human retinal pigment epithelial cells *in vitro*: evaluation of necrosis, apoptosis, cell migration, cell proliferation and expression of tissue repairing and cytoprotective genes. *PLoS ONE*.

[103] Gao Y.-X., Yu C.-A., Lu J.-H. (2013). ADAMTS-7 expression increases in the early stage of angiotensin II-induced renal injury in elderly mice. *Kidney and Blood Pressure Research*.

[104] Dunn J. R., Reed J. E., du Plessis D. G. (2006). Expression of ADAMTS-8, a secreted protease with antiangiogenic properties, is downregulated in brain tumours. *British Journal of Cancer*.

[105] Moriguchi-Goto S., Yamashita A., Tamura N. (2009). ADAMTS-13 attenuates thrombus formation on type I collagen surface and disrupted plaques under flow conditions. *Atherosclerosis*.

[106] Collins-Racie L. A., Flannery C. R., Zeng W. (2004). ADAMTS-8 exhibits aggrecanase activity and is expressed in human articular cartilage. *Matrix Biology*.

[107] Ocak Z., Acar M., Gunduz E. (2013). Effect of hypericin on the ADAMTS-9 and ADAMTS-8 gene expression in MCF7 breast cancer cells. *European Review for Medical and Pharmacological Sciences*.

[108] Demircan K., Hirohata S., Nishida K. (2005). ADAMTS-9 is synergistically induced by interleukin-1*β* and tumor necrosis factor *α* in OUMS-27 chondrosarcoma cells and in human chondrocytes. *Arthritis and Rheumatism*.

[109] Dubail J., Aramaki-Hattori N., Bader H. L. (2014). A new *Adamts9* conditional mouse allele identifies its non-redundant role in interdigital web regression. *Genesis*.

[110] Sengle G., Tsutsui K., Keene D. R. (2012). Microenvironmental regulation by fibrillin-1. *PLoS Genetics*.

[111] Fontanil T., Rúa S., Llamazares M. (2014). Interaction between the ADAMTS-12 metalloprotease and fibulin-2 induces tumor-suppressive effects in breast cancer cells. *Oncotarget*.

[112] Moncada-Pazos A., Obaya A. J., Llamazares M. (2012). ADAMTS-12 metalloprotease is necessary for normal inflammatory response. *The Journal of Biological Chemistry*.

[113] El Hour M., Moncada-Pazos A., Blacher S. (2010). Higher sensitivity of Adamts12-deficient mice to tumor growth and angiogenesis. *Oncogene*.

[114] Johnston P., Chojnowski A. J., Davidson R. K., Riley G. P., Donell S. T., Clark I. M. (2007). A complete expression profile of matrix-degrading metalloproteinases in Dupuytren's disease. *Journal of Hand Surgery*.

[115] Rodriguez-Lopez J., Pombo-Suarez M., Loughlin J. (2009). Association of a nsSNP in ADAMTS14 to some osteoarthritis phenotypes. *Osteoarthritis and Cartilage*.

[116] Poonpet T., Honsawek S., Tammachote N., Kanitnate S., Tammachote R. (2013). ADAMTS14 gene polymorphism associated with knee osteoarthritis in Thai women. *Genetics and Molecular Research*.

[117] El Khoury L., Posthumus M., Collins M., Handley C. J., Cook J., Raleigh S. M. (2013). Polymorphic variation within the ADAMTS2, ADAMTS14, ADAMTS5, ADAM12 and TIMP2 genes and the risk of Achilles tendon pathology: a genetic association study. *Journal of Science and Medicine in Sport*.

[118] Goertsches R., Comabella M., Navarro A., Perkal H., Montalban X. (2005). Genetic association between polymorphisms in the *ADAMTS14* gene and multiple sclerosis. *Journal of Neuroimmunology*.

[119] Molokwu C. N., Adeniji O. O., Chandrasekharan S., Hamdy F. C., Buttle D. J. (2010). Androgen regulates ADAMTS15 gene expression in prostate cancer cells. *Cancer Investigation*.

[120] Peluffo M. C., Murphy M. J., Baughman S. T., Stouffer R. L., Hennebold J. D. (2011). Systematic analysis of protease gene expression in the rhesus macaque ovulatory follicle: metalloproteinase involvement in follicle rupture. *Endocrinology*.

[121] Stupka N., Kintakas C., White J. D. (2013). Versican processing by a disintegrin-like and metalloproteinase domain with thrombospondin-1 repeats proteinases-5 and -15 facilitates myoblast fusion. *The Journal of Biological Chemistry*.

[122] Demircan K., Topcu V., Takigawa T. (2014). ADAMTS4 and ADAMTS5 knockout mice are protected from versican but not aggrecan or brevican proteolysis during spinal cord injury. *BioMed Research International*.

[123] Gao S., De Geyter C., Kossowska K., Zhang H. (2007). FSH stimulates the expression of the ADAMTS-16 protease in mature human ovarian follicles. *Molecular Human Reproduction*.

[124] Pyun J.-A., Kim S., Kwack K. (2014). Interaction between thyroglobulin and *ADAMTS16* in premature ovarian failure. *Clinical and Experimental Reproductive Medicine*.

[125] Pyun J.-A., Kim S., Cha D. H., Kwack K. (2014). Epistasis between polymorphisms in TSHB and ADAMTS16 is associated with premature ovarian failure. *Menopause*.

[126] Morales J., Al-Sharif L., Khalil D. S. (2009). Homozygous mutations in ADAMTS10 and ADAMTS17 cause lenticular myopia, ectopia lentis, glaucoma, spherophakia, and short stature. *The American Journal of Human Genetics*.

[127] van Duyvenvoorde H. A., Lui J. C., Kant S. G. (2014). Copy number variants in patients with short stature. *European Journal of Human Genetics*.

[128] Arning A., Hiersche M., Witten A. (2012). A genome-wide association study identifies a gene network of ADAMTS genes in the predisposition to pediatric stroke. *Blood*.

[129] Aldahmesh M. A., Khan A. O., Mohamed J. Y. (2011). Identification of ADAMTS18 as a gene mutated in Knobloch syndrome. *Journal of Medical Genetics*.

[130] Jin H., Wang X., Ying J. (2007). Epigenetic identification of ADAMTS18 as a novel 16q23.1 tumor suppressor frequently silenced in esophageal, nasopharyngeal and multiple other carcinomas. *Oncogene*.

[131] Peluso I., Conte I., Testa F. (2013). The ADAMTS18 gene is responsible for autosomal recessive early onset severe retinal dystrophy. *Orphanet Journal of Rare Diseases*.

[132] Li Z., Nardi M. A., Li Y.-S. (2009). C-terminal ADAMTS-18 fragment induces oxidative platelet fragmentation, dissolves platelet aggregates, and protects against carotid artery occlusion and cerebral stroke. *Blood*.

[133] Wei J., Liu C.-J., Li Z. (2014). Adamts-18: a metalloproteinase with multiple functions. *Frontiers in Bioscience*.

[134] Knauff E. A. H., Franke L., van Es M. A. (2009). Genome-wide association study in premature ovarian failure patients suggests ADAMTS19 as a possible candidate gene. *Human Reproduction*.

[135] Cal S., Obaya A. J., Llamazares M., Garabaya C., Quesada V., López-Otín C. (2002). Cloning, expression analysis, and structural characterization of seven novel human ADAMTSs, a family of metalloproteinases with disintegrin and thrombospondin-1 domains. *Gene*.

[136] Pyun J.-A., Kim S., Cha D. H., Kwack K. (2013). Epistasis between *IGF2R* and *ADAMTS19* polymorphisms associates with premature ovarian failure. *Human Reproduction*.

[137] Pyun J.-A., Kim S., Kwack K. (2015). Epistasis between polymorphisms in ACVR2B and ADAMTS19 is associated with premature ovarian failure. *Menopause*.

[138] Silver D. L., Hou L., Somerville R., Young M. E., Apte S. S., Pavan W. J. (2008). The secreted metalloprotease ADAMTS20 is required for melanoblast survival. *PLoS Genetics*.

